# Association between Continuity of Care and the Onset of Thyroid Disorder among Diabetes Patients in Korea

**DOI:** 10.3390/ijerph16020233

**Published:** 2019-01-15

**Authors:** Sang Ah Lee, Sung-Youn Chun, Woorim Kim, Yeong Jun Ju, Dong-Woo Choi, Eun-Cheol Park

**Affiliations:** 1 Department of Public Health, Graduate School, Yonsei University, Seoul 03722, Korea; ivory0817@yuhs.ac (S.A.L.); wklaura@gmail.com (W.K.); cdw6027@yuhs.ac (D.-W.C.); 2 Institute of Health Services Research, Yonsei University, Seoul 03722, Korea; csy6909@yuhs.ac (S.-Y.C.); joomeon@gmail.com (Y.J.J.); 3 Department of community health science, University of Nevada Las Vegas, Las Vegas, NV 89154-9900, USA; 4 Department of Preventive Medicine and Public Health, Ajou University, School of Medicine, Suwon 16499, Korea; 5 Department of Preventive Medicine, Yonsei University College of Medicine, Seoul 03722, Korea

**Keywords:** diabetes, continuity of care, thyroid disorder, complication, comorbidity

## Abstract

*Objectives*: As the relationship between diabetes mellitus and thyroid dysfunction is well known, it is important to investigate the factors influencing this association. Continuity of care is associated with better quality of care and outcomes, such as reduced complications, among diabetes patients. Therefore, the purpose of this study was to investigate the association between continuity of care and the onset of thyroid dysfunction among diabetes patients. *Methods*: We used Korean National Health Insurance Service National Sample Cohort data from 2002 to 2013. Our final study population included 16,806 newly diagnosed diabetes patients who were older than 45 years of age. Continuity of care was measured using the Continuity of Care index. The dependent variable was the onset of thyroid disorder. Cox proportional hazard regression models were used for statistical analyses. *Results*: Diabetes patients with low continuity of care were at increased risk of the onset of thyroid disorder compared with those with high continuity of care (hazard ratio (HR): 1.28, 95% confidence interval (CI): 1.07–1.54). Subgroup analyses showed that this association was significant within patients with type 2 diabetes (HR: 1.24, 95% CI: 1.01–1.52) or whose main attending site was a local clinic (HR: 1.32, 95% CI: 1.07–1.64). *Conclusions*: Our results show that diabetes patients with low continuity of care are more likely to experience the onset of thyroid disorder. Therefore, improving continuity of care could be a reasonable method of preventing complications or comorbidities, including thyroid disorder, among diabetes patients.

## 1. Introduction

Diabetes is a common chronic disease that cannot be easily treated and causes severe complications. This severe non-communicable disease has been increasing in prevalence worldwide. According to the International Diabetes Federation, three people are newly diagnosed with diabetes every 10 seconds globally; additionally, it is predicted that 10% of people will suffer from diabetes by 2030 [1]. Diabetes is a metabolic disorder that can cause serious secondary complications such as blindness, non-traumatic lower extremity amputation, and terminal renal failure, resulting in massive burdens for family members and society [2].

Additionally, diabetes is associated with diverse comorbidities such as obesity, dyslipidemia, and hypertension [3]. Of the various comorbidities associated with diabetes, some studies have focused on the association between diabetes and thyroid function [4,5]. The relationship between diabetes mellitus and thyroid dysfunction has been known for many years [5,6], and they have shown mutual influence [7]. In addition, patients with diabetes have a higher prevalence of thyroid diseases than the general population [8]. Diabetes seems to influence thyroid function by affecting the conversion mechanism of T4 to T3 in the peripheral tissue, and influencing hypothalamic control of thyroid stimulating hormone release [9,10]. As unmanaged diabetes may induce abnormal thyroid hormone [11], continuous monitoring of diabetes patients is imperative for screening for and prevention of thyroid disorders. In this context, continuity of care (COC) could be an important method due to its association with positive outcomes such as preventing complications among diabetes patients [12].

COC is described as a continuous partnership between physician and patient [13] and is associated with better quality of care [14]. COC can be achieved by knowing how a patient with a specific past history progresses through medical care with the same physician or at the same institution [15]. Breaking of COC is known to be associated with negative outcomes. Peterson et al. suggest that discontinuity of care, such as patient transfer to another physician, is associated with adverse events and that communication between those physicians might decrease adverse events [16,17]. In other words, COC has a positive effect on treatment. Previous studies report that COC has various positive effects such as improved patients treatment adherence [14,18] and better outcomes [19]. Particularly, COC is known to improve the quality of care of people with chronic diseases [20,21]. Among diabetes patients, continuous care is required to control blood glucose levels and provide better quality of care, with several previous studies suggesting that diabetes complications can be delayed or prevented by controlling blood glucose level [22,23].

Therefore, the purpose of this study was to investigate the association between COC and the onset of thyroid dysfunction among newly diagnosed diabetes patients. As the Korean government encourages the use of local clinics for managing chronic diseases such as hypertension and diabetes, we also examined whether the association between COC and the onset of thyroid disorder differs depending on the type of diabetes and main attending site.

## 2. Methods

### 2.1. Data and Study Population

We used Korean National Health Insurance Service National Sample Cohort (NHIS-NSC) data from 2002 to 2013. This dataset includes all medical claims from 1,025,340 individuals representing 2% of the South Korean population. For this study, we used only outpatient data from patients aged 45 years or older which were considered a risky group for diabetes [24]. We first excluded patients who had diabetes in 2002 to 2003 to extract new onset of diabetes. We included patients who used outpatient services for treating their diabetes mellitus more than 4 times in the first 2 years after their diagnosis date. We excluded patients who died in the first 2 years after their diabetes diagnosis date, who had thyroid disorder before the onset of diabetes, or who experienced the onset of thyroid disorder within the first 2 years after their diabetes diagnosis date. Our final study population included 16,806 individuals. NHIS-NSC data is secondary data and do not contain any data which can identify individuals. Therefore, ethical approval is exempted. The requirement for informed consent was waived because the study was based on routinely collected administrative or claims data.

### 2.2. Variables

The COC index considers not only the frequency of visits to providers but also the distribution of visits between providers [15,25]. The index ranges from 0 to 1. A score of 1 indicates that all visits are made to the same provider, whereas a score of 0 indicates that all visits are made to different providers. In this study, level of COC was divided into low-group and high-group based on 0.75 as the cut-off point [26]. COC was calculated as:

(1) $$\mathrm{COC}=\frac{\sum_{i=1}^{M}n_{i}^{2}-N}{N\left(N-1\right)}$$

where *N* is total number of visits, $n_{i}$ is number of visits to provider *i*, and *M* is the total number of providers.

Our dependent variable was the new onset of thyroid disorder (hypothyroidism or hyperthyroidism, ICD (International Classification of Diseases) codes ‘E03’ or ‘E05’, respectively) during the least 2 years after the onset of diabetes.

We controlled patients’ age (45–54, 55–64, 65–74, or 75+ years), gender, income quartile (Q1 for lowest to Q4 for highest), insurance type (National Health Insurance or Medical Aid), residential area (metropolitan area or non-metropolitan area), type of diabetes (type 1 or 2), Charlson Comorbidity Index, presence of disability, main attending site (general hospital, hospital, or local clinic), location of provider (capital area, metropolitan area, or non-metropolitan area), foundation of provider (public, corporation, or private), and diabetes onset year.

### 2.3. Statistical Analysis

Pearson’s Chi-squared tests were used to examine general characteristics of the study population by testing for differences in the distribution of each variable. Cox proportional hazard regression models were used to compute hazard ratios (HRs) for the effect of COC on the onset of thyroid disorder. Log-rank tests were conducted after verifying that there were no violations of the proportional hazards assumption. We also conducted subgroup analyses stratified by type of diabetes and type of main attending site to investigate whether these factors influenced the association between COC and the onset of thyroid dysfunction.

## 3. Results

Table 1 shows the general characteristics of the study population. Among 16,806 diabetes patients, 3.0% (*n* = 504) experienced the onset of thyroid disorder, whereas 97.0% (*n* = 16,302) did not. Regarding COC, 69.7% (*n* = 11,717) experienced high COC, whereas 30.3% (*n* = 5089) experienced low COC. Regarding type of diabetes, 13.8% (*n* = 2314) had type 1 diabetes, whereas 86.2% (*n* = 14,492) had type 2 diabetes. Regarding main attending site, 69.5% (*n* = 11,671) used a local clinic, 22.5% (*n* = 3787) used a general hospital, and 8.0% (*n* = 1348) used a hospital. Mean COC was 0.829 ± 0.237 for, 0.802 ± 0.248, and 0.830 ± 0.237 for all patients, patients in the thyroid disorder group, and patients in the no thyroid disorder group, respectively.

Table 2 shows factors associated with the onset of thyroid disorder among diabetes patients. Patients with low COC were at increased risk of the onset of thyroid disorder compared with those with high COC (HR: 1.28, 95% confidence interval (CI): 1.07–1.54). Also, the risk of onset of thyroid disorder decreased with increasing age. Patients aged 75+ years showed the lowest rate of onset of thyroid disorder compared with patients aged 45–54 years (HR: 0.45, 95% CI: 0.30–0.68). Furthermore, women had a higher risk of onset of thyroid disorder than men (HR: 2.31, 95% CI: 1.87–2.85).

Table 3 shows the results of subgroup analyses stratified by type of diabetes and main attending site. Regarding type of diabetes, a significant association between COC and onset of thyroid disorder was found among patients with type 2 diabetes (HR: 1.24, 95% CI: 1.01–1.52) but not among patients with type 1 diabetes. Regarding main attending site, a significant association between COC and onset of thyroid disorder was found only for local clinics (HR: 1.32, 95% CI: 1.07–1.64) but not for general hospitals or hospitals.

## 4. Discussion

In this study, we investigated the association between COC and the onset of thyroid disorders among diabetes mellitus patients in Korea. We found that individuals with low COC showed a higher risk of thyroid disorder onset compared with those with high COC. This association was found among patients with type 2 diabetes or who visited a local clinic as their main attending site.

Insufficient glucose control causes several complications or comorbidities among patients with diabetes, and continuous control of blood glucose is imperative for preventing or delaying diabetes complications or comorbidities [23]. Previous studies showed that uncontrolled glucose also affects thyroid hormones [27,28]. Therefore, appropriate glucose control of diabetes patients should be achieved for preventing onset of thyroid disorder among patients with diabetes. In this process, continuity of care could have a positive effect on continuous blood glucose control.

Generally, COC has been recognized for having various positive effects on care processes, such as enhanced communication between physician and chronic disease patients [29] and improved likelihood of keeping follow-up appointments [30]. Additionally, provider continuity has positive effects on the quality of care and produces better outcomes due to the formation of long-term relationships and accumulation of knowledge between patients and providers [31]. Thus, patients with high COC tend to have better glycemic control and more well-managed diabetes [32]. Several studies show that diabetes patients with high COC have a lower risk of onset of complications [33,34]. Even though one previous study showed that patients with high COC did not show statistically significant association with thyroid outpatients visit, the study confirmed high COC was associated with reduced diabetes ketoacidosis [35]. In addition, Knight et al. [36] showed that patients with diabetes showed lower rate of hospitalization due to chronic diseases including thyroid disorders when their continuity of care was high. Therefore, high COC may be related to well-controlled glycemic status, which could help prevent the onset of thyroid disorders.

Our subgroup analyses showed statistically significant associations between COC and the onset of thyroid disorder among type 2 diabetes. Several previous studies reported the relationship between type 2 diabetes and thyroid disorders [37,38,39]. Considering this association, continuity of care could be one of the methods for preventing the onset of thyroid disorder among patients with diabetes. We also found that diabetes patients whose main attending site was a local clinic showed a statistically significant association between COC and the onset of thyroid disorder, whereas those whose main attending site was a hospital or general hospital did not show a significant association. This finding may be due to systematic differences among types of sites, such as physicians’ rotation systems, consultation hours, characteristics of patients, and number of patients per physicians. Further research is needed to determine why an association between COC and the onset of thyroid disorder was found only for local clinics.

Our study has several limitations. First, we could not adjust other important covariates, including educational level, working status, physical activity level or family history, which could be associated with the onset of thyroid disorder. Second, the severity of diabetes was not included in our dataset. Third, we defined thyroid disorder based on ICD-10 codes made when patients visited clinics and were diagnosed with thyroid disorder; therefore, we could not detect subclinical thyroid disorder. Fourth, we did not separate thyroid disorders into hyperthyroidism and hypothyroidism; therefore, further study is needed to focus on each type of thyroid disorder. Fifth, the primary purpose of this data was health insurance claims, and the accuracy of administrative data has been discussed for several years [40]. However, a previous study which studied the accuracy of this data demonstrated a 70% accuracy [41]. Despite these limitations, our study has the strength of using nationwide claims data obtained from the National Health Insurance Service. Additionally, to the best of our knowledge, there are few studies on the association between COC and the onset of thyroid disorder among diabetes patients [35,36]. Therefore, our study could add to the body of evidence of the association.

## 5. Conclusions

Diabetes is a common non-communicable disease, and its management is crucial for preventing many complications and comorbidities. Considering that diabetes and thyroid disorders are the most prevalent endocrine diseases, our study shows that diabetes patients with low COC were more likely to experience the onset of thyroid disorder compared to patients with high COC. High COC is associated with better quality of care and diabetes management as well as protection from complications. Thus, COC could be a reasonable method of preventing complications or comorbidities, including thyroid disorder, among diabetes patients. As “medical shopping” is a healthcare problem in Korea, preventing patients from medical shopping and enhancing their COC is needed.

## Figures and Tables

**Table 1:** The general characteristics of the study population.

Variables	Total		The Onset of Thyroid Disorder				*p*-Value
			No		Yes		
	*N*	(%)	*N*	(%)	*N*	(%)	
Continuity of care index							0.0017
High (≥0.75)	11,717	69.7	11,398	97.3	319	2.7	
Low (<0.75)	5089	30.3	4904	96.4	185	3.6	
Age group							0.0066
45~54	5748	34.2	5567	96.9	181	3.2	
55~64	5501	32.7	5315	96.6	186	3.4	
65~74	4024	23.9	3914	97.3	110	2.7	
75+	1533	9.1	1506	98.2	27	1.8	
Gender							<0.0001
Male	9282	55.2	9095	98.0	187	2.0	
Female	7524	44.8	7207	95.8	317	4.2	
Income							
Low	3445	20.5	3372	97.9	73	2.1	
Middle	8738	52.0	8478	97.0	260	3.0	
High	4623	27.5	4452	96.3	171	3.7	
Insurance type							0.1183
National Health Insurance	15,895	94.6	15,410	97.0	485	3.1	
Medical aids	911	5.4	892	97.9	19	2.1	
Residential area							0.0716
Capital area	6926	41.2	6697	96.7	229	3.3	
Metropolitan area	4272	25.4	4143	97.0	129	3.0	
Rural area	5608	33.4	5462	97.4	146	2.6	
Type of Diabetes Mellitus							0.0641
Type 1	2314	13.8	2230	96.4	84	3.6	
Type 2	14,492	86.2	14,072	97.1	420	2.9	
Charlson comorbidity index							
2+	14,803	88.1	14,328	96.8	475	3.2	
1	1571	9.4	1547	98.5	24	1.5	
0	432	2.6	427	98.8	5	1.2	
Disability							0.1745
No	14,977	89.1	14,51	96.9	459	3.1	
Yes	1829	10.9	1784	97.5	45	2.5	
Main attending clinic							0.6649
General hospital	3787	22.5	3672	97.0	115	3.0	
Hospital	1348	8.0	1313	97.4	35	2.6	
Clinic	11,671	69.5	11,317	97.0	354	3.0	
Location of the main attending clinic							0.2921
Capital area	6865	40.9	6646	96.8	219	3.2	
Metropolitan area	4483	26.7	4346	96.9	137	3.1	
Rural area	5458	32.5	5310	97.3	148	2.7	
Foundation of the main attending clinic							0.7098
Public	1606	9.6	1561	97.2	45	2.8	
Corporation	3870	23.0	3747	96.8	123	3.2	
Private	11,330	67.4	10,994	97.0	336	3.0	
Diabetes onset year							<0.0001
2004	2466	14.7	2324	94.2	142	5.8	
2005	2309	13.7	2195	95.1	114	4.9	
2006	1832	10.9	1773	96.8	59	3.2	
2007	1980	11.8	1915	96.7	65	3.3	
2008	1983	11.8	1930	97.3	53	2.7	
2009	2085	12.4	2045	98.1	40	1.9	
2010	1866	11.1	1849	99.1	17	0.9	
2011	2285	13.6	2271	99.4	14	0.6	
Total	16,806	100.0	16,302	97.0	504	3.0	

**Table 2:** The factors associated with the onset of thyroid disorder among diabetes mellitus patients by cox proportional hazard regression.

Variables	The Onset of Thyroid Disorder		
	HR	95% CI	*p*-Value
Continuity of care (COC) index			
High (≥0.75)	1.00	-	
Low (<0.75)	1.28	(1.07–1.54)	0.0078
Age group			
45~54	1.00	-	
55~64	0.94	(0.76–1.15)	0.5247
65~74	0.66	(0.52–0.85)	0.0012
75+	0.45	(0.30–0.68)	0.0001
Gender			
Male	1.00	-	
Female	2.31	(1.87–2.85)	<0.0001
Income			
Low	1.00	-	
Middle	1.11	(0.87–1.43)	0.4047
High	1.42	(1.12–1.81)	0.0037
Insurance type			
Supporter	1.00	-	
Dependent	1.05	(0.84–1.30)	0.6854
Residential area			
Capital area	1.00	-	
Metropolitan area	0.76	(0.48–1.18)	0.2180
Rural area	0.66	(0.44–1.00)	0.0515
Type of diabetes mellitus			
Type 1	1.00	-	
Type 2	1.02	(0.80–1.29)	0.8931
Charlson comorbidity index			
0	1.00	-	
1	0.93	(0.72–1.20)	0.5754
2+	1.07	(0.83–1.37)	0.6160
Disability			
Yes	1.00	-	
No	0.97	(0.71–1.32)	0.8416
Main attending clinic			
General hospital	1.00	-	
Hospital	0.99	(0.65–1.51)	0.9709
Clinic	1.24	(0.85–1.80)	0.2611
Location of the main attending clinic			
Capital area	0.79	(0.52–1.21)	0.2809
Metropolitan area	0.96	(0.64–1.45)	0.8560
Rural area	1.00	-	
Foundation of the main attending clinic			
Public	1.00	-	
Corporation	1.40	(0.90–2.16)	0.1328
Private	1.12	(0.81–1.54)	0.4924
Diabetes onset year			
2004	1.00	-	
2005	0.93	(0.73–1.20)	0.5860
2006	0.71	(0.52–0.96)	0.0280
2007	0.90	(0.66–1.23)	0.5155
2008	0.88	(0.63–1.23)	0.4529
2009	0.87	(0.60–1.27)	0.4802
2010	0.71	(0.42–1.20)	0.1995
2011	1.35	(0.74–2.45)	0.3237

HR: hazard ratio; CI: confidence interval.

**Table 3:** The results of subgroup analyses of the association between the continuity of care and the onset of thyroid disorder stratified by the type of diabetes mellitus and main attending clinic.

Variables	The Onset of Thyroid Disorder			
	High COC	Low COC		
	HR	HR	95% CI	*p*-Value
Type of diabetes mellitus *				
Type 1	1.00	1.46	(0.95–2.24)	0.0864
Type 2	1.00	1.24	(1.01–1.52)	0.0382
Main attending clinic **				
General hospital	1.00	1.13	(0.75–1.68)	0.5641
Hospital	1.00	1.46	(0.73–2.90)	0.2817
Clinic	1.00	1.32	(1.07–1.64)	0.0107

* Adjusted for age, gender, income, insurance type, residential area, Charlson Comorbidity Index, disability existence, main attending clinic, location of the main attending clinic, and foundation of the main attending clinic, diabetes onset year. ** Adjusted for age, gender, income, insurance type, residential area, Charlson Comorbidity Index, disability existence, type of diabetes mellitus, location of the main attending clinic, and foundation of the main attending clinic, diabetes onset year.
